# Supplementary material for: Rapid Dynamic Naturalistic Monitoring of Bradykinesia in Parkinson’s Disease Using a Wrist-Worn Accelerometer
Source: Sensors (Basel). 2021 Nov 26;21(23):7876. doi: 10.3390/s21237876 (PMC8659489; doi:10.3390/s21237876)
Supplement: Supplementary file 1 [file sensors-21-07876-s001.zip › sensors-1463464-supplementary.pdf]

Supplemental Material Naturalistic rapid PD bradykinesia monitoring Habets et al

Table S1: Overview of extracted features. All features were extracted over x-, y-, z-, axes, and signal vector magnitude signals. Literature source shows first author name, see reference list for full reference.

**Feature name**	**Source/ literature**
Temporal domain	
Maximum acc	Griffiths[1]
Interquartile range of acc	Griffiths[1]
90th percentile of acc	Rispens [2]
median of acc	Hoff [3,] Keijsers [4]
mean of acc	Hoff [3,] Keijsers [4]
standard deviation of acc	Shawen [5]
coefficient of variation	Shawen [5]
variance of acc	Shawen [5]
acceleration range	Mahadevan [6]
low acc peaks (n)	Balasubramanian [7]
high acc peaks (n)	Balasubramanian [7]
time spent above 1g acc (%)	Keijsers [4,] Salarian [8]
acc entropy	Mahadevan [6,] Lonini [9]
jerkiness ratio/ smoothness	Mahadevan [6,] Lonini [9]
root mean square (RMS)	Mahadevan [6]

Ratio of x/y/z-RMS compared to vector magnitude-RMS	Sekine [10]
Axial cross-correlation (X-Y; (X-Z,; Y-Z)	Mahadevan [6,] Lonini [9]
Spectral domain	
spectral power < 3.5 Hz	Griffiths[1,] Evers [11]
spectral power 0.7 < 1.4 Hz	Griffiths[1,] Evers [11]
spectral power 1.4 < 2.8 Hz	Griffiths[1,] Evers [11]
spectral power 2.8 < 3.5 Hz	Griffiths[1,] Evers [11]
spectral flatness	Mahadevan [6]
spectral entropy	Mahadevan [6]
spectral variance	Balasubramanian [7,] Beck [12]
spectral smoothness	Balasubramanian [7]
spectral low/high peaks	Balasubramanian [7]
Dominant frequency magnitude	Mahadevan [6,] Lonini [9]
Dominant frequency ratio	Mahadevan [6]
Dominant frequency flatness	Mahadevan [6]
Dominant frequency entropy	Mahadevan [6]

Figure S1: **Schematic visualization of data splitting method for individual models.**
Pre- and post-medication features were balanced in number.
During every fold in individual-model training and testing, ⅕ of pre-medication data and ⅕ of post-medication data (two times 10% of total data leads to 20% of total data), were selected as ‘test data’ for the validation of the model which was trained using the ‘training data’. For the training data selection, blocks of 2% of data adjacent to the test data were excluded, to decrease the temporal dependence of the training and test data.
Due to the expected difference in activities between blocks of 10% of data, we repeated model training and testing with every consecutive block of 10% of data. This led to 41 different training and testing folds. This figure visualizes folds 1, 2, 3, 18, 32, 40, and 41 as examples.

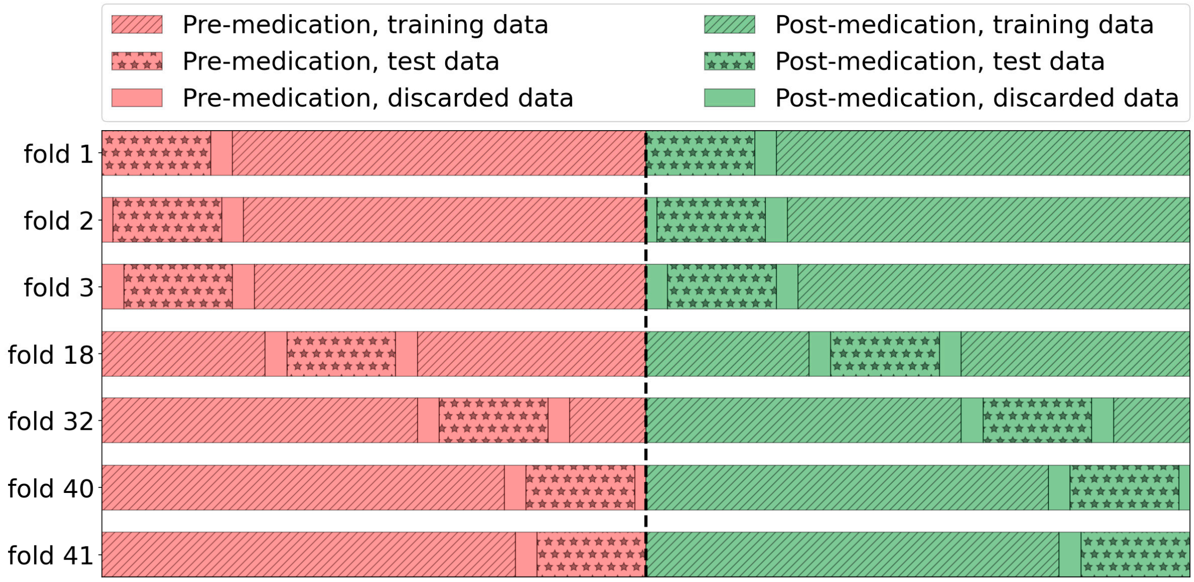

Figure S2: Visualization of activity filter performance versus the parallel raw signal vector magnitude.

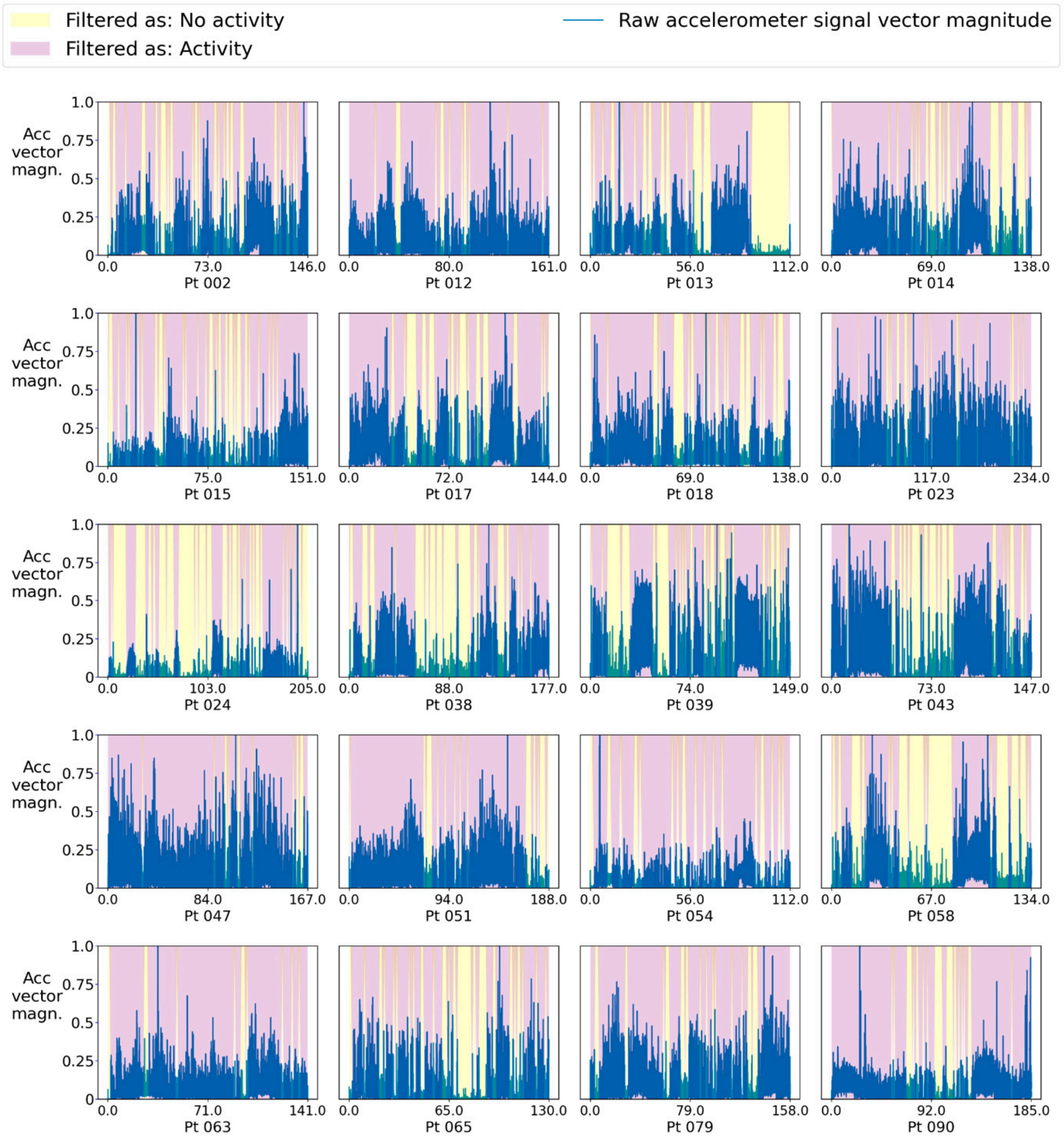

Y-axes represent the vector magnitude of the raw tri-axial accelerometer signal (in blue). The accelerometer vector magnitudes are individually normalized with a maximum of 1.0 for visualization purposes since maximum acceleration vectors vary between individuals.
X-axes represent time in minutes, where the first half consists of pre-medication recordings, and the second half of post-medication recordings.
The background color-shading represents whether the activity filtered classified the data epoch as activity present (purple), or not present (yellow).

Table S2: Predictive metrics for all models and approaches

			All features	All features	4 features	4 features
CLASSIFIER		n=20, mean (sd)	All minutes	Activity filtered	All minutes	Activity filtered
SUPPORT VECTOR	*INDIVIDUAL MODEL*	*auc*	0.682 (0.15)	0.696 (0.18)	0.499 (0.12)	0.533 (0.16)
		auroc, n sign	16	13	2	4
		accuracy	0.632 (0.12)	0.651 (0.14)	0.490 (0.11)	0.509 (0.14)
		accuracy, n sign	15	14	5	10
	GROUP MODEL	*auroc*	0.669 (0.10)	0.703 (0.10)	0.590 (0.11)	0.633 (0.13)
		auroc, n sign	16	17	10	11
		accuracy	0.624 (0.09)	0.640 (0.08)	0.560 (0.11)	0.597 (0.08)
		accuracy, n sign	11	12	9	12
RANDOM FOREST	*INDIVIDUAL MODEL*	*auroc*	0.649 (0.13)	0.656 (0.17)	0.586 (0.12)	0.619 (0.14)
		auroc, n sign	15	10	6	11
		accuracy	0.611 (0.10)	0.611 (0.13)	0.558 (0.10)	0.588 (0.11)
		accuracy, n sign	13	10	8	7
	GROUP MODEL	*auroc*	0.661 (0.10)	0.698 (0.11)	0.593 (0.11)	0.636 (0.14)
		auroc, n sign	12	16	10	10

		accuracy	0.598 (0.08)	0.626 (0.08)		0.564 (0.08)	0.587 (0.08)
		accuracy, n sign	12	12		6	7

Figure S3: Comparison of different model approaches for short window medication states classification

To understand which methodological approaches yielded best performance, we explored the differences between individual and group trained models and the effect of an activity filter in detail and investigated optimal training data sizes and feature window lengths (see Methods section in main text). For this we compared the 20 areas under the receiver operator characteristic (AUC) and classification accuracy scores of each model using equality plots. Each dotted line visualizes the line x = y, and represents equality of the two displayed models.

The p-values throughout these figures indicate whether the ratio of patients that scored higher on model X versus model Y is statistically significant. We performed a 5000 permutation test where 20 dots (random x-value, random y-value) were randomly plotted in the equality plot. The p-values represent the chance that the distribution is better than the random chance level.

Figures S3A and S3B show classification superiority of models analyzing 60 seconds data epochs using 103 accelerometer-derived features compared to 4 features (AUC p-values < 0.002, accuracy p-values < 0.020, significance tested via a 5000-permutation test).
The consecutive S3C-F figures only display the models including 103 features. Individual models based on a support vector classifier (SV) resulted in higher AUC scores and accuracies than individual models based on random forest classifier (RF) in 15 out of 20 patients (figure S3C, p = 0.009 below). SV and RF group models yielded similar AUC scores and accuracies (figure S3D below, p = 0.406). Overall, applying the activity filter led to slightly better mean results per model (table S2). On an individual level, there was no significant difference between classification performance with or without activity filtering (figure S3EF, p-values ranged between 0.06 and 0.41). However, it was noted that there was a trend towards higher individual predictive performance with activity filtering.

Figure S3A: Individual models: 4 versus 103 features.

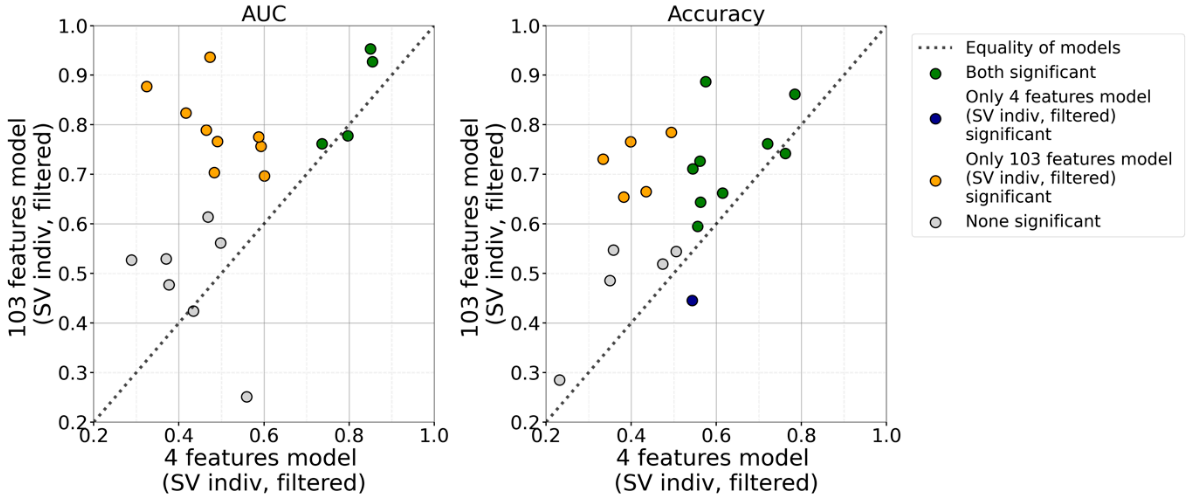

Individual 103 features model higher than individual 4 features model for:
AUC: 17 out of 20 higher, p < 0.000, accuracy: 18 out of 20 higher: p < 0.000

Figure S3B: Group models: 4 versus 103 features.

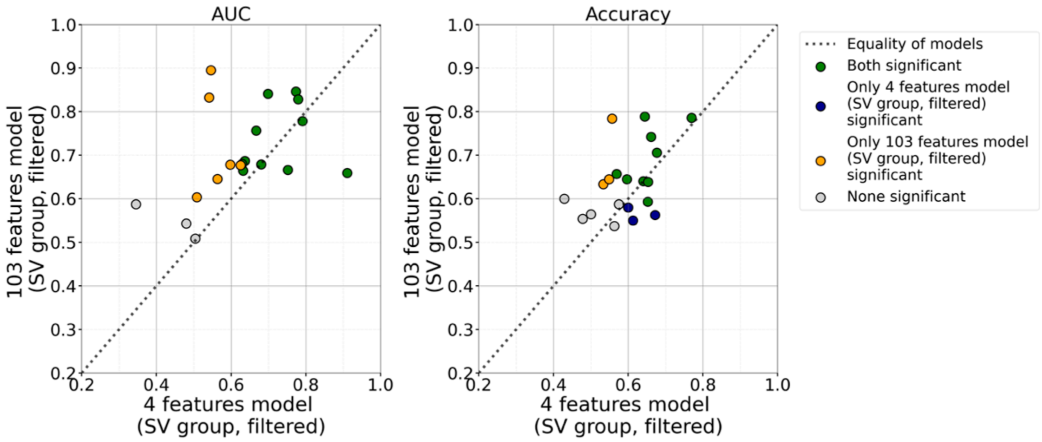

Group 103 features model higher than group 4 features model for:
AUC: 16 out of 20 higher, p = 0.002, accuracy: 14 out of 20 higher: p = 0.023

Figure S3C: Individual models: SV versus RF classifiers.

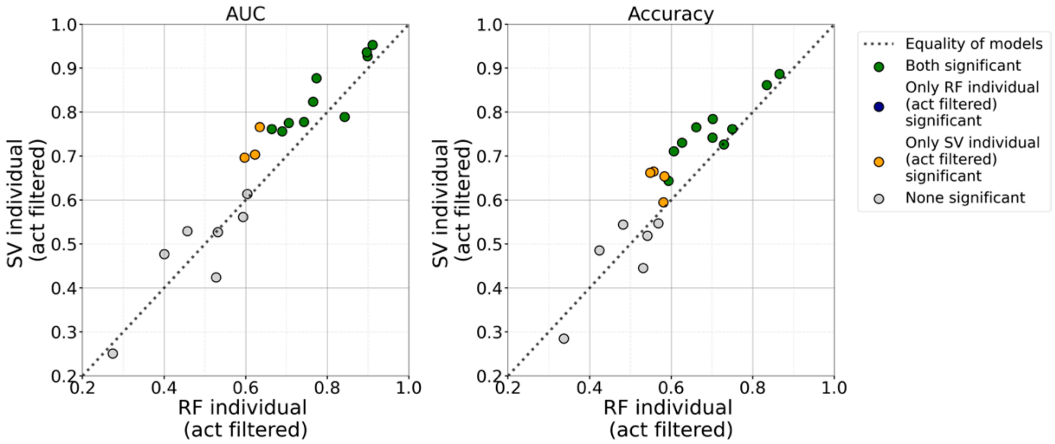

Individual SV model higher than individual RF model for:
AUC: 15 out of 20 higher, p = 0.009, accuracy: 15 out of 20 higher: p = 0.009

Figure S3D: Group models: SV versus RF classifiers.

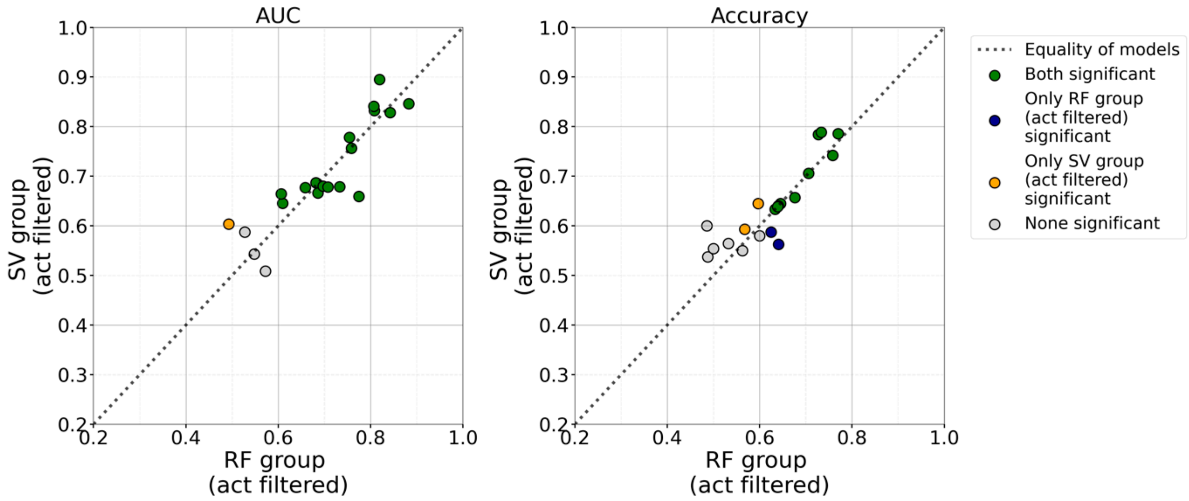

Group SV model higher than group RF model for:
AUC: 10 out of 20 higher, p = 0.406, accuracy: 10 out of 20 higher: p = 0.406

Figure S3E: Individual SV models: with activity filtering versus without activity filtering.

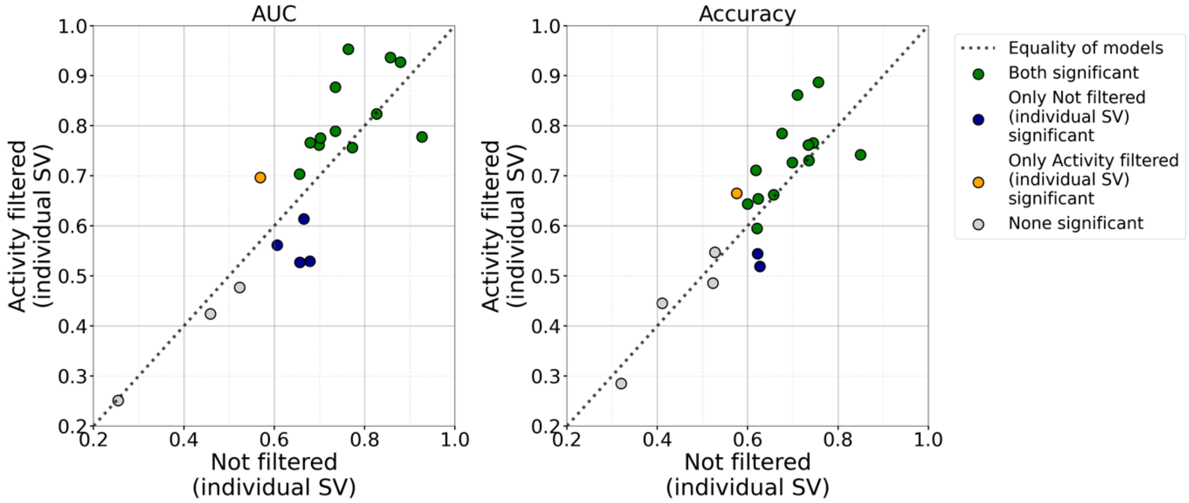

Individual SV model with activity filtering higher than individual SV model without activity filtering for:
AUC in 10 out of 20 higher, p = 0.406, accuracy in 13 out of 20 higher, p = 0.056.

Note that both the AUC scores and the classification accuracies of the activity filtered models are marginally higher than those of the not filtered models, when all ‘none significant’ candidates are disregarded. We conclude that although there is no statistically significant superiority of the activity filtered models, there is a trend that activity filtered models lead to higher predictive performance and this step was included within our standard pipeline.

Figure S3F: Group SV models: with activity filtering versus without activity filtering.

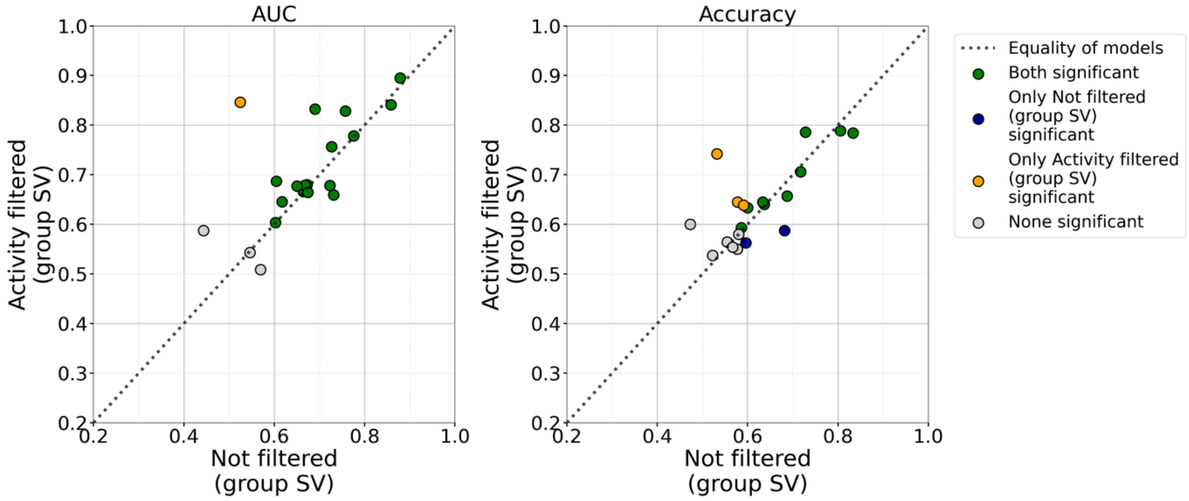

Group SV model with activity filtering higher than group SV model without activity filtering for:
AUC in 13 out of 20 higher, p = 0.056, accuracy in 11 out of 20 higher, p = 0.250.

Figure S4A: Good classification performance in patients with and without tremor

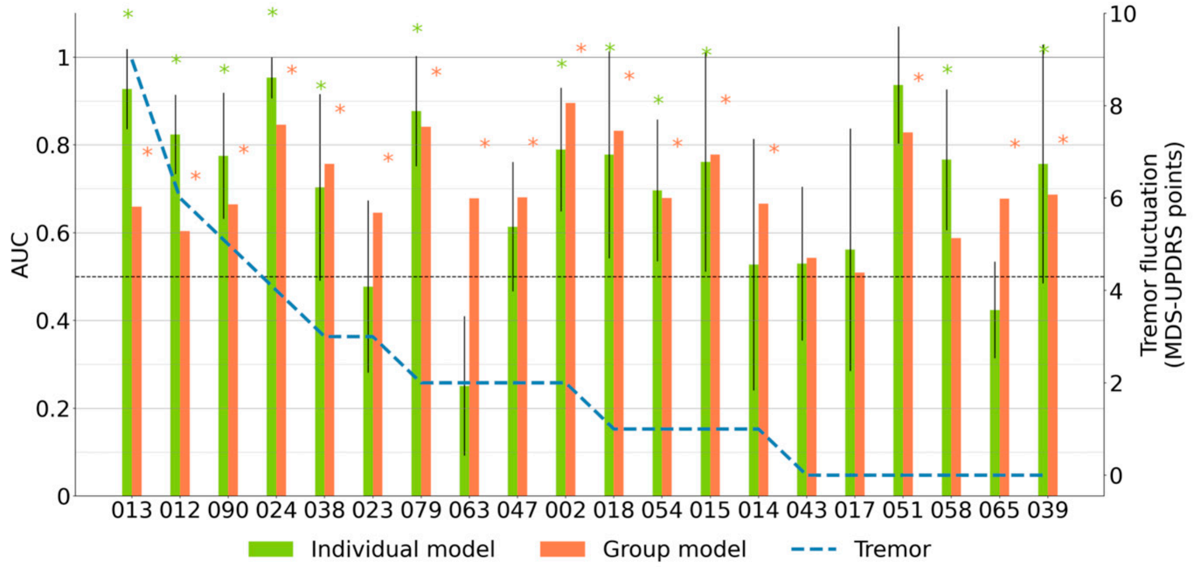

Figure S4B: Good classification performance in patients with and without abnormal involuntary movements

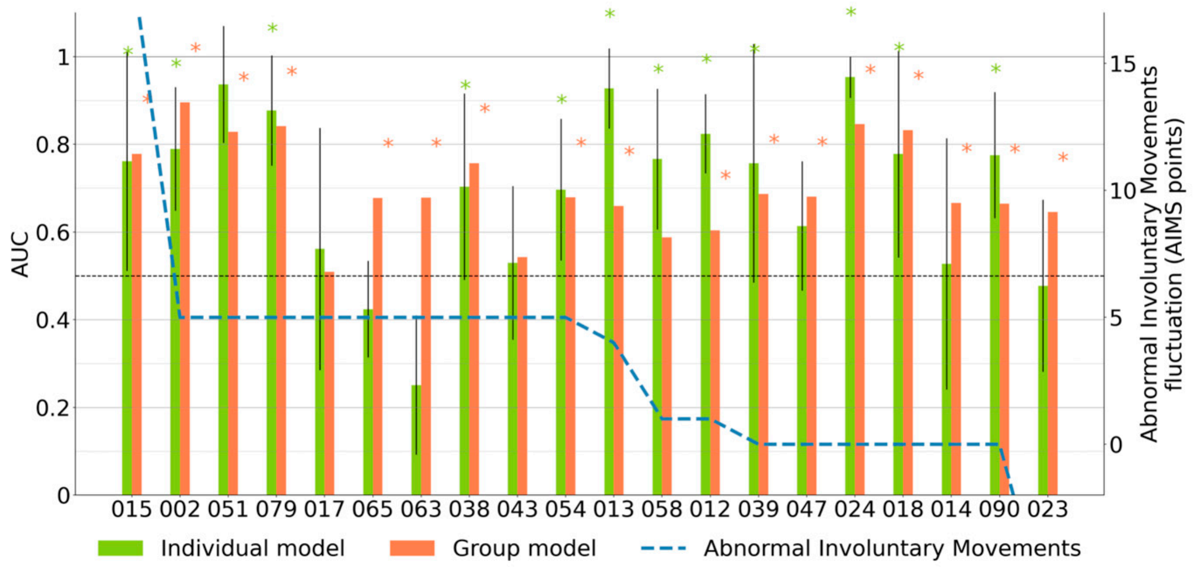

Colored bars visualize the individual AUC scores from the best individual and the best group model (both support vector classifier, and activity filtered), and correspond to the left y-axis. Individual tremor fluctuations between pre- and post-medication correspond to the right y-axis. Tremor scores represent the described MDS-UPDRS III items for unilateral upper extremity tremor (see Methods). On the x-axis individual participants are sorted on tremor fluctuation, in descending order. Colored asterisks indicate statistical significance of the AUC score compared to chance level (alpha = 0.05, FDR corrected). The black dotted line indicates chance-level for the AUC scores. AUC: area under the receiver operator characteristic; FDR: false discovery rate, MDS-UPDRS: Movement Disorders Society - Unified Parkinson Disease Rating Scale.

Table S3: Spearman R correlations between symptom fluctuation and predictive performance at an individual level. Spearman r correlations are calculated between the MDS-UPDRS tremor and bradykinesia and AIMS fluctuations (collected and calculated as described in the Methods), and the predictive performance per participant. Support vector models including activity filtering were compared for individual and group model comparisons.

	Individual models, AUC	Group models, AUC	Individual models, accuracy	Group models, accuracy
Bradykinesia (r (p))	0.24 (p = 0.305)	0.01 (p = 0.962)	0.24 (p = 0.305)	0.18 (p = 0.452)
Tremor (r (p))	0.34 (p = 0.140)	0.11 (p = 0.642)	0.21 (p = 0.380)	-0.06 (p = .807)
Abnormal involuntary movements (r (p))	-0.05 (p = 0.851)	0.122 (p = 0.607)	0.01 (p = 0.979)	0.34 (p = 0.142)

References

1. Griffiths, R. I. *et al.* Automated assessment of bradykinesia and dyskinesia in Parkinson's disease. *J Park. Dis* **2**, 47–55 (2012).
2. Rispens, S. M. *et al.* Identification of fall risk predictors in daily life measurements: gait characteristics' reliability and association with self-reported fall history. *Neurorehabil. Neural Repair* **29**, 54–61 (2015).
3. Hoff, J. I., van der Meer, V. & van Hilten, J. J. Accuracy of objective ambulatory accelerometry in detecting motor complications in patients with Parkinson disease. *Clin Neuropharmacol* **27**, 53–7 (2004).
4. Keijsers, N. L., Horstink, M. W. & Gielen, S. C. Ambulatory motor assessment in Parkinson's disease. *Mov Disord* **21**, 34–44 (2006).
5. Shawen, N. *et al.* Role of data measurement characteristics in the accurate detection of Parkinson's disease symptoms using wearable sensors. *J. NeuroEngineering Rehabil.* **17**, 52 (2020).
6. Mahadevan, N. *et al.* Development of digital biomarkers for resting tremor and bradykinesia using a wrist-worn wearable device. *NPJ Digit Med* **3**, 5 (2020).
7. Balasubramanian, S., Melendez-Calderon, A. & Burdet, E. A robust and sensitive metric for quantifying movement smoothness. *IEEE Trans. Biomed. Eng.* **59**, 2126–2136 (2012).
8. Salarian, A. *et al.* Quantification of tremor and bradykinesia in Parkinson's disease using a novel ambulatory monitoring system. *IEEE Trans Biomed Eng* **54**, 313–22 (2007).
9. Lonini, L. *et al.* Wearable sensors for Parkinson's disease: which data are worth collecting for training symptom detection models. *Npj Digit. Med.* **1**, 64 (2018).
10. Sekine, M. *et al.* A gait abnormality measure based on root mean square of trunk acceleration. *J. NeuroEngineering Rehabil.***10**, 118 (2013).
11. Evers, L. J. *et al.* Real-Life Gait Performance as a Digital Biomarker for Motor Fluctuations: The Parkinson@Home Validation Study. *J Med Internet Res* **22**, e19068 (2020).
12. Beck, Y. *et al.* SPARC: a new approach to quantifying gait smoothness in patients with Parkinson's disease. *J. NeuroEngineering Rehabil.* **15**, 49 (2018).
